# Modeling cerebrospinal fluid dynamics across the entire intracranial space through integration of four-dimensional flow and intravoxel incoherent motion magnetic resonance imaging

**DOI:** 10.1186/s12987-024-00552-6

**Published:** 2024-05-30

**Authors:** Shigeki Yamada, Tomohiro Otani, Satoshi Ii, Hirotaka Ito, Chifumi Iseki, Motoki Tanikawa, Yoshiyuki Watanabe, Shigeo Wada, Marie Oshima, Mitsuhito Mase

**Affiliations:** 1 Department of Neurosurgery, Nagoya City University Graduate School of Medical Science, Kawasumi 1, Mizuho-Cho, Mizuho-Ku, Nagoya, Aichi 467-8601 Japan; 2 Interfaculty Initiative in Information Studies/Institute of Industrial Science, The University of Tokyo, Tokyo, Japan; 3 Department of Mechanical Science and Bioengineering, Graduate School of Engineering Science, Osaka University, Osaka, Japan; 4 Department of Mechanical Engineering, School of Engineering, Tokyo Institute of Technology, Tokyo, Japan; 5 Faculty of System Design, Tokyo Metropolitan University, Tokyo, Japan; 6 Medical System Research & Development Center, FUJIFILM Corporation, Tokyo, Japan; 7 Department of Behavioural Neurology and Cognitive Neuroscience, Tohoku University Graduate School of Medicine, Sendai, Miyagi Japan; 8 Division of Neurology and Clinical Neuroscience, Department of Internal Medicine III, Yamagata University School of Medicine, Yamagata, Japan; 9 Department of Radiology, Shiga University of Medical Science, Shiga, Japan

**Keywords:** Cerebrospinal fluid dynamics, Idiopathic normal pressure hydrocephalus, Aging, Hakim’s disease, Idiopathic normal pressure hydrocephalus, Aging, CSF motion, Fluid oscillation, 4D Flow MRI, Intravoxel incoherent motion, Diffusion-weighted image

## Abstract

**Background:**

Bidirectional reciprocal motion of cerebrospinal fluid (CSF) was quantified using four-dimensional (4D) flow magnetic resonance imaging (MRI) and intravoxel incoherent motion (IVIM) MRI. To estimate various CSF motions in the entire intracranial region, we attempted to integrate the flow parameters calculated using the two MRI sequences. To elucidate how CSF dynamics deteriorate in Hakim’s disease, an age-dependent chronic hydrocephalus, flow parameters were estimated from the two MRI sequences to assess CSF motion in the entire intracranial region.

**Methods:**

This study included 127 healthy volunteers aged ≥ 20 years and 44 patients with Hakim’s disease. On 4D flow MRI for measuring CSF motion, velocity encoding was set at 5 cm/s. For the IVIM MRI analysis, the diffusion-weighted sequence was set at six b-values (i.e., 0, 50, 100, 250, 500, and 1000 s/mm^2^), and the biexponential IVIM fitting method was adapted. The relationships between the fraction of incoherent perfusion (*f*) on IVIM MRI and 4D flow MRI parameters including velocity amplitude (VA), absolute maximum velocity, stroke volume, net flow volume, and reverse flow rate were comprehensively evaluated in seven locations in the ventricles and subarachnoid spaces. Furthermore, we developed a new parameter for fluid oscillation, the Fluid Oscillation Index (FOI), by integrating these two measurements. In addition, we investigated the relationship between the measurements and indices specific to Hakim’s disease and the FOIs in the entire intracranial space.

**Results:**

The VA on 4D flow MRI was significantly associated with the mean *f*-values on IVIM MRI. Therefore, we estimated VA that could not be directly measured on 4D flow MRI from the mean *f*-values on IVIM MRI in the intracranial CSF space, using the following formula; *e*^0.2(*f*−85)^ + 0.25. To quantify fluid oscillation using one integrated parameter with weighting, FOI was calculated as VA × 10 + *f* × 0.02. In addition, the FOIs at the left foramen of Luschka had the strongest correlations with the Evans index (Pearson’s correlation coefficient: 0.78). The other indices related with Hakim’s disease were significantly associated with the FOIs at the cerebral aqueduct and bilateral foramina of Luschka. FOI at the cerebral aqueduct was also elevated in healthy controls aged ≥ 60 years.

**Conclusions:**

We estimated pulsatile CSF movements in the entire intracranial CSF space in healthy individuals and patients with Hakim’s disease using FOI integrating VA from 4D flow MRI and *f*-values from IVIM MRI. FOI is useful for quantifying the CSF oscillation.

**Supplementary Information:**

The online version contains supplementary material available at 10.1186/s12987-024-00552-6.

## Background

The dynamics of cerebrospinal fluid (CSF) have been demonstrated in many imaging studies and animal models, but remains mysterious. Complex CSF motions comprise a steady microflow produced by the rhythmic wavy movement of motile cilia on the ventricular wall surface [1, 2]; dynamic multidirectional pulsatile, laminar, and turbulent flows produced by pulsations of the brain and cerebral arteries [2–8], and an uncertain flow produced by respiration and head movement [9–12]. However, measuring very small complex CSF motions with a velocity of < 0.1 cm/s had been difficult using conventional phase-contrast magnetic resonance imaging (MRI) [4, 13] and four-dimensional (4D) flow MRI [6, 7, 14–16]. Furthermore, previous CSF dynamic studies have measured pulsatile CSF motions in the ventricles, including the foramen of Magendie, cerebral aqueduct, and foramina of Monro, and subarachnoid spaces in the posterior fossa, including the prepontine cistern and craniocervical junction, but not in the Sylvian fissures or the convexity part of the subarachnoid spaces [3–8, 13–16]. Furthermore, these complex pulsatile CSF motions have been considered to be mainly driven by arterial pulsations [10, 15, 17, 18] and decrease with aging because of declines in brain volume, arterial elasticity, and circulating cerebral blood volume [6, 8, 19, 20]. These age-related changes in CSF dynamics and volumes, including metabolism, are of interest because they are thought to be associated with dementia and neurodegenerative diseases by impairing the excretion of neurotoxic wastes from the brain [18, 21–23]. Moreover, tightened sulci in high convexity [24–26] have been recognized as the most important imaging finding for Hakim’s disease, which has been called idiopathic normal-pressure hydrocephalus [27] in contrast to brain atrophy in the elderly population. Accumulating evidence supports the hypothesis that various alterations in CSF dynamics contribute to disproportionately enlarged subarachnoid spaces and ventricles in Hakim’s disease, which is considered an age-dependent chronic hydrocephalus. We previously reported the usefulness of the mean value of fraction of incoherent perfusion (*f*) on intravoxel incoherent motion (IVIM) MRI for evaluating small complex CSF motions in the entire intracranial CSF space, comparing brains with Hakim’s disease with healthy aging brains [28], although their direction and flow velocity could not be examined on IVIM MRI [29–31]. Le Bihan et al. described visualization of CSF motion in their first paper on IVIM MRI [29], and the possibility that *f* values could quantify CSF motion in their second paper [30]. Therefore, we hypothesized that complex CSF motions in the entire intracranial space could be simulated using flow velocity parameters on 4D flow MRI and *f* on IVIM MRI. Confirming this hypothesis may contribute to the elucidation of CSF dynamics in Hakim’s disease and aging effects. Therefore, this study aimed to estimate CSF motion from dynamic pulsatile flow to fine microflow by integrating *f* on IVIM MRI and flow velocity parameters on 4D flow MRI. In addition, our second purpose is to elucidate how alterations in CSF dynamics contribute to enlarged subarachnoid spaces and ventricles in Hakim’s disease, using high-resolution 3D morphological models of the ventricles and subarachnoid spaces in the entire intracranial space. Furthermore, this study compared the distribution of CSF motions in healthy aging volunteers with that in patients with Hakim’s disease.

## Methods

### Study population

In total, 128 healthy volunteers aged ≥ 20 years who underwent both 4D flow MRI and IVIM MRI on a 3-T brain MRI scanner (Discovery MR 750W, GE Medical Systems, Inc. Chicago, IL, USA) from November 2020 to February 2022 were recruited from the medical staff, students, and their family members by open recruitment. The inclusion criteria for the volunteers were as follows: individuals who had no history of brain injury, brain tumor, or cerebrovascular disease on previous brain MRI and those who had never undergone brain MRI and had no neurological symptoms, including cognitive function. One volunteer aged 84 years was excluded from this study because he had a history of head surgery due to a head injury over 30 years ago. Patients’ MRI data were used in an opt-out method, after their personal information was anonymized in a linkable manner. Furthermore, 25 patients diagnosed with Hakim’s disease who underwent MRI using the same GE MRI machine as that in the volunteers until 2022 and 19 patients with Hakim’s disease who underwent MRI using a Philips MRI machine (Ingenia Elition 3.0 T Philips, Amsterdam, Netherlands) since 2023 were included in this study. All patients with Hakim’s disease had radiological findings of disproportionately enlarged subarachnoid space hydrocephalus (DESH) [32–34], specifically ventricular dilatation, enlarged Sylvian fissure, and narrow sulci at high convexity [26], and triad symptoms of gait disturbance, cognitive impairment, and urinary incontinence, according to the third edition of the Japanese guidelines for the management of idiopathic normal-pressure hydrocephalus [35]. Of them, 26 patients (59%) underwent CSF removal (30–35 mL) via a lumbar tap and were evaluated for changes in their symptoms before and 1 and 2 days after the CSF tap test. Furthermore, 25 patients (57%) underwent CSF shunt surgery, and their symptoms improved by ≥ 1 point on the modified Rankin Scale and/or the Japanese grading scale [35]. Other patients were not recommended to undergo an aggressive tap test or shunt surgery because their symptoms were very mild or they could not walk, and no improvement in symptoms was observed after the tap test or shunt surgery.

### Image acquisition

The sequence parameters for 4D flow MRI using a GE MRI machine were as follows: repetition time, variable (10–20 ms); echo time, variable (3–7 ms); flip angle, 8°; field of view, 200 mm; matrix size, 256 × 256; voxel size, 0.781 × 0.781 × 1.0 mm; number of cardiac phases, 12; and velocity encoding, 5 cm/s. The 4D flow sequence parameters for a Philips MRI machine were as follows: repetition time, variable (10–20 ms); echo time, variable (3–7 ms); flip angle, 8°; field of view, 200 mm; matrix size, 208 × 198; voxel size, 0.89 × 0.89 × 1.0 mm; number of cardiac phases, 8; and velocity encoding, 5 cm/s. The image range was in the mid-sagittal plane with a width of 30 mm (1.0 mm × 30 slices), encompassing areas from the bilateral foramina of Monro to the upper cervical subarachnoid spaces. The imaging time was approximately 10 min, depending on the individual heart rate, which was synchronized with the peripheral pulse rate measured from the finger. Because the original T1-weighted magnitude images, including the 4D flow sequence set, did not have sufficient resolution, the 3D T2-weighted fast spin-echo sequence was alternatively performed to obtain additional anatomical information, with the following parameters: repetition time, 2000 ms; echo time, 85.3 ms; matrix 288 × 288; voxel size, 0.8 × 0.8 × 0.8 mm; and acquisition time, approximately 4 min.

For the IVIM analysis, diffusion-weighted imaging was performed in the axial plane using six b values (i.e., 0, 50, 100, 250, 500, and 1000 s/mm^2^). The sequence parameters of a GE MRI machine were as follows: repetition time, variable (6000–7000 ms); echo time, variable (75–80 ms); flip angle, 90°; slice thickness, 3.0 mm; field of view, 220 mm; acquisition matrix size, 128 × 192; pixel size, 1.7 × 1.1 mm; acquisition time, approximately 2 min for total acquisition; motion-probing gradient, bipolar type. The IVIM sequence parameters for a Philips MRI machine were as follows: repetition time, variable (10–20 ms); echo time, variable (3–7 ms); flip angle, 90°; slice thickness, 3.0 mm; field of view, 220 mm; acquisition matrix size, 123 × 192; pixel size, 0.98 × 0.98 mm. The isotropic images were created from three orthogonal diffuse gradient pulse images.

### 4D flow MRI analysis

**Fig. 1:**
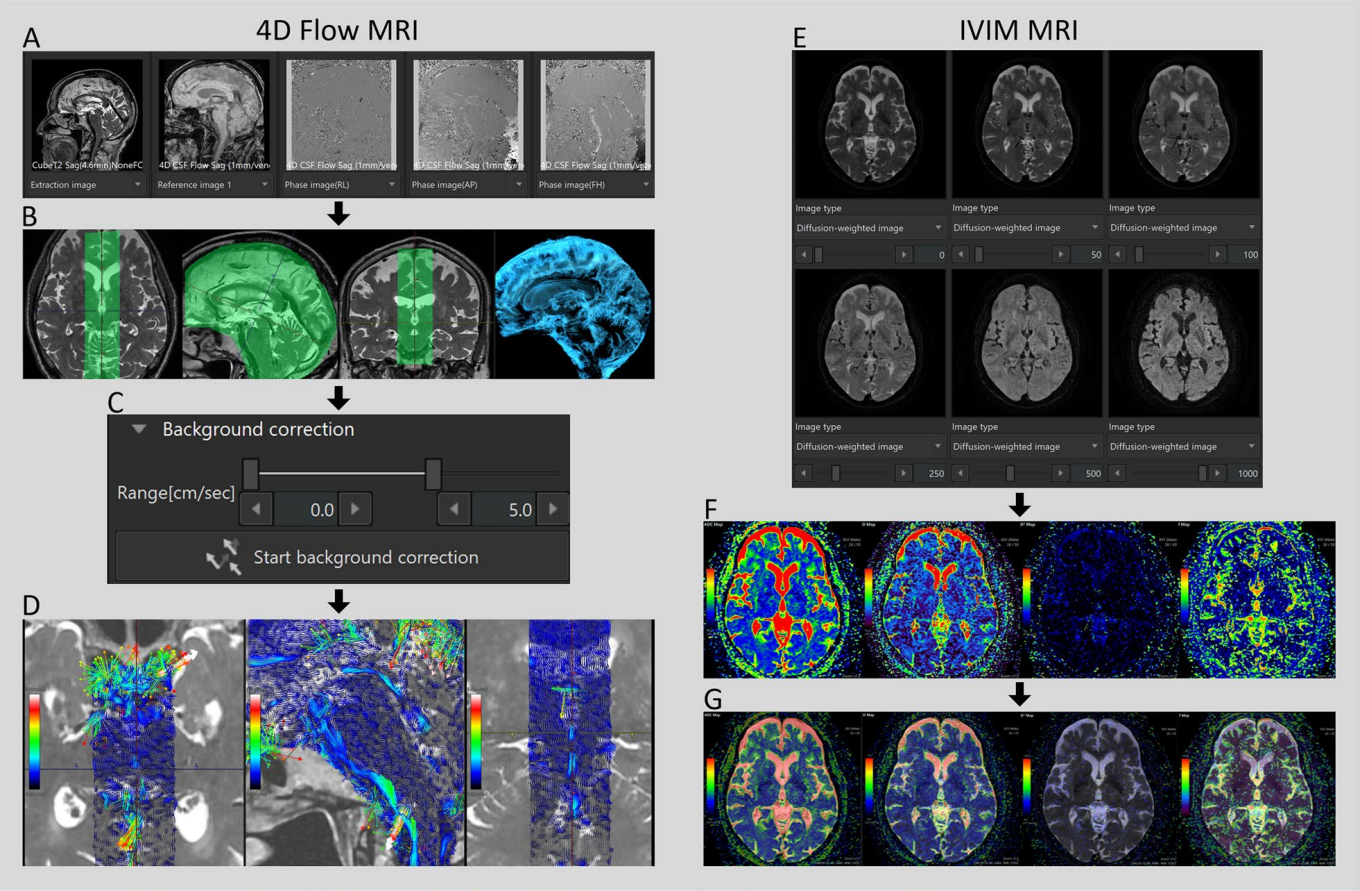
Schema of our methods of 4D flow MRI and intravoxel incoherent motion (IVIM) MRI. The figures on the left illustrate (**A**–**D**) the 4D Flow MRI, while those on the right (**E**–**G**) depict the IVIM MRI, both acquired from a single healthy volunteer aged 85 years. **A** Demonstrates the initiation of the 4D Flow app. Four-dimensional analysis is performed utilizing the 3D T2-weighted MRI (leftmost), the morphological image of the 4D flow MRI (second from the left) as the reference, and tri-axis phase images (right three). Following the extraction of the area designated for 4D flow analysis (**B**, light green), background correction is executed (**C**), which is fully automated with a single click. Finally, an observation phase is undertaken to evaluate CSF flow velocity (**D**). In **E**, the IVIM app has just been launched, and all six low b-values of DWI images (0, 50, 100, 250, 500, and 1000 s/mm^2^) are integrated. The ADC map (leftmost), D map (second left), D* map (second right), and *f* map (rightmost) are automatically and instantly calculated (**F**). **G** Displays a b = 0 DWI image automatically overlaid on four maps

Details of the acquisition method for 4D flow MRI were described in our previous papers [6, 7, 20]. In summary, in the first step of 4D flow MRI, 3D velocity encoding data obtained from triaxial phase-contrast images of 4D flow MRI and morphological data of the intracranial CSF space obtained from 3D T2-weighted MRI were combined using a commercialized 4D flow application on an independent 3D volume analyzer workstation (SYNAPSE 3D; FUJIFILM Corporation, Tokyo, Japan), as shown in Fig. 1. In the second step, the area to be analyzed by 4D flow MRI was extracted. In the third step, automated polynomial fitting properly was used to correct for phase offsets, eddy currents and background noise, enabling accurate measurements of slow CSF flow velocities [36]. Finally, the 3D flow velocity vectors, including the average, maximum, and minimum values of triaxial velocities (cm/s), stroke volume (μL/heartbeat), net flow volume (μL/heartbeat), and reverse flow rate (%), were measured at nine regions of interest (ROIs) based on anatomical features: ventral and dorsal aspects of the foramen magnum, foramen of Magendie, lower and upper parts of the cerebral aqueduct, bilateral foramina of Monro, premedullary cistern, and prepontine cistern on the orthogonal planes perpendicular to the mid-sagittal section and perpendicular to the main axis of CSF motion. Because the CSF moves bidirectionally, velocity amplitude (VA) (cm/s) was defined as the difference between the maximum velocity and the minimum velocity (typically the maximum velocity in the opposite direction) at each ROI.

### Intravoxel incoherent motion analysis

IVIM analysis to quantitatively evaluate small pulsatile complex CSF motions was performed as previously reported [28]. In brief, from the six low b-values of DWI images (0, 50, 100, 250, 500, and 1000 s/mm^2^), the commercialized IVIM application on the SYNAPSE 3D workstation creates *f* maps, concurrently with other parameters, including apparent diffusion coefficient (ADC, mm^2^/s), true diffusion coefficient (D, mm^2^/s), and pseudo-diffusion coefficient (D*, mm^2^/s), in one voxel automatically and instantly (Fig. 1F).

We adapted the biexponential IVIM fitting method using the Levenberg–Marquardt algorithm with a constraint on *f* (0 < *f* < 1) as following equation:

$$\frac{S}{S0}=f\cdot {\text{exp}}\left(-{{\text{bD}}}^{*}\right)+\left(1-f\right)\cdot {\text{exp}}\left(-{\text{bD}}\right)$$

where* S* is the signal intensity at a given b value, *S*0 is the signal intensity at b = 0 s/mm^2^. After IVIM analysis, b = 0 DWI images were automatically superimposed on the *f* maps as shown in Fig. 1G. The average, maximum, and minimum values of *f* were measured in the following 45 ROIs based on anatomical features: the foramen magnum; bilateral foramina of Luschka; foramen of Magendie; fourth ventricle; lower and upper parts of the cerebral aqueduct; anterior and posterior parts of the third ventricle; bilateral foramina of Monro; anterior horn, body, trigone, and inferior horn of the bilateral lateral ventricles; bilateral cerebellopontine angle; prepontine cistern; interpeduncular cistern; lamina terminalis cistern; suprasellar or chiasmatic cistern; quadrigeminal cistern; bilateral ambient cisterns; bilateral carotid cisterns; anterior and posterior rami of the bilateral Sylvian sulci; bilateral Sylvian fossae; lower and upper parts of the interhemispheric fissure; bilateral superior frontal sulci; bilateral central sulci; and bilateral marginal sulci.

### Fluid oscillation index

After comprehensive evaluation of the relationship between parameters on 4D flow MRI and *f*-values on IVIM MR, we developed a new parameter for fluid oscillation, the Fluid Oscillation Index (FOI), by integrating these two measurements.

### Measurements and indices specific to Hakim’s disease

The total ventricles and subarachnoid spaces were manually segmented from the 3D T2-weighted cube sequence using our original method combined with a simple threshold algorithm and manual segmentation, as previously reported [33, 34, 37]. Furthermore, the area and maximum anteroposterior diameter of the foramen of Magendie and the maximum anteroposterior diameter of the bilateral foramina of Luschka were measured. The indices specific to Hakim’s disease—the Evans index defined as the maximal width of the frontal horns of the lateral ventricles to the maximal width of the internal diameter of the cranium based on the x-dimension; Z-Evans index defined as the maximum z-axial length of the frontal horns of the lateral ventricles to the maximum cranial z-axial length on the coronal plane, which was perpendicular to the anteroposterior commissure plane on the anterior commissure [33]; brain per ventricle ratios (BVRs) defined as the maximum width of the brain just above the lateral ventricles divided by the maximum width of the lateral ventricles on the reference coronal planes at the anterior commissure and posterior commissure levels, respectively [34]; and callosal angle defined as the angle of the roof of the bilateral ventricles on the coronal plane at the posterior commissure level [38]—were measured.

### Statistical analysis

The volunteers were divided into the following three subgroups according to their ages at the time of MRI examination: < 40 years, 40–59 years, and ≥ 60 years. The mean ± standard deviation (SD) for several measurements and indices in the three age subgroups were compared using the Kruskal–Wallis rank sum test, and those in patients with Hakim’s disease and healthy elderly aged ≥ 60 years were compared using the Mann–Whitney–Wilcoxon test. The chi-square test was used to compare the proportions of the groups. The relationship between the mean *f*-values on IVIM MRI and flow parameters measured on 4D flow MRI, such as VA, absolute maximum velocity, mean velocity, stroke volume, net flow volume, and reverse flow rate at the following seven ROIs: left and right foramina of Monro, upper and lower parts of the cerebral aqueduct, foramen of Magendie, dorsal part of the foramen magnum, and prepontine cistern. These parameters were investigated using Pearson’s correlation coefficient (*r*) and 95% confidential intervals (CIs). The ROI of the lower part of the cerebral aqueduct for 4D flow MRI was treated as the ROI of the upper part of the fourth ventricle for IVIM MRI. In addition, we investigated the relationship between the measurements and indices specific to Hakim’s disease and the FOIs in the entire intracranial space. Statistical significance was assumed at a probability (*P*) value of < 0.05. All missing data points were treated as deficit data that did not affect other variables. Statistical analyses were performed using R (version 4.2.3; The R Foundation for Statistical Computing; http://www.R-project.org).

## Results

### Clinical characteristics

In this study, 127 healthy volunteers (< 40 years, n = 45; 40–59 years, n = 46; ≥ 60 years, n = 36), including 44 males and 83 females, and 44 Hakim’s patients (77.1 ± 7.0 years), including 28 males and 16 females, were included. The linear and volumetric indices for the size of the intracranial CSF space, including the ventricular systems, among the three age groups of healthy volunteers and patients with Hakim’s disease are summarized in Table 1. Significant differences in all measurements were observed between patients with Hakim’s disease and healthy elderly individuals aged ≥ 60 years.

**Table 1:** Clinical characteristics of the study population

	Healthy volunteers			Hakim patients	*P*1	*P*2
	< 40 years	40–59 years	≥ 60 years			
Total number	45	46	36	44		
Sex (female:male)	30:15	32:14	21:15	28:16	0.071	0.550
Age (years)	29.2 ± 5.4	49.4 ± 5.8	69.2 ± 6.9	77.1 ± 7.0	< 0.001	< 0.001
Evans index	0.25 ± 0.02	0.25 ± 0.02	0.26 ± 0.02	0.37 ± 0.18	< 0.001	0.045
Z-Evans index	0.24 ± 0.03	0.24 ± 0.03	0.27 ± 0.04	0.43 ± 0.14	< 0.001	< 0.001
BVR at AC	2.1 ± 0.3	2.1 ± 0.3	1.8 ± 0.4	0.83 ± 0.27	< 0.001	< 0.001
BVR at PC	4.8 ± 1.4	4.3 ± 1.1	3.0 ± 1.0	0.99 ± 0.22	< 0.001	< 0.001
Callosal angle (degree)	119.9 ± 9.4	118.7 ± 11.4	117.0 ± 11.5	68.9 ± 16.7	< 0.001	0.614
Area of the foramen of Magendie	13.3 ± 9	11.3 ± 5.8	18.5 ± 10.5	41.8 ± 21.6	< 0.001	0.002
Diameter of the foramen of Magendie	3.1 ± 1.4	2.8 ± 1	3.5 ± 1.2	6.7 ± 2.5	< 0.001	0.104
Diameter of the right foramen of Luschka	1.4 ± 0.8	1.7 ± 0.9	1.7 ± 1.0	3.4 ± 1.5	< 0.001	0.290
Diameter of the left foramen of Luschka	1.7 ± 0.9	2.3 ± 1.2	2.2 ± 1.2	3.7 ± 1.4	0.002	0.014
Total ventricles (mL)	19.5 ± 7.5	22 ± 8.2	36.4 ± 14.3	127 ± 33.5	< 0.001	< 0.001
Total ventricles (%)	1.3 ± 0.5	1.5 ± 0.5	2.5 ± 0.9	8.4 ± 1.8	< 0.001	< 0.001
Total subarachnoid spaces (mL)	267 ± 35.7	287 ± 43.2	352 ± 55.0	325 ± 44.6	0.032	< 0.001
Total subarachnoid spaces (%)	18.2 ± 2	20.1 ± 2.3	24.5 ± 2.7	21.6 ± 2.5	< 0.001	< 0.001
Total intracranial CSF space (mL)	287 ± 39.1	309 ± 47.8	388 ± 64.7	452 ± 56.2	< 0.001	< 0.001
Total intracranial CSF space (%)	19.5 ± 2.2	21.6 ± 2.4	27.0 ± 3.0	30.0 ± 2.5	< 0.001	< 0.001

*P*1; probability value for the Mann–Whitney–Wilcoxon test between patients with Hakim’s disease and healthy volunteers aged ≥ 60 years

*P*2; probability value for the Kruskal–Wallis test among the three age groups of healthy volunteers

Sex differences were compared using Fisher’s exact test

*AC* anterior commissure, *PC* posterior commissure, *CSF* cerebrospinal fluid, *BVR* brain per ventricle ratio, *AC* anterior commissure, *PC* posterior commissure

### Relationship between flow parameters on 4D flow MRI and f-values on IVIM MRI

**Fig. 2:**
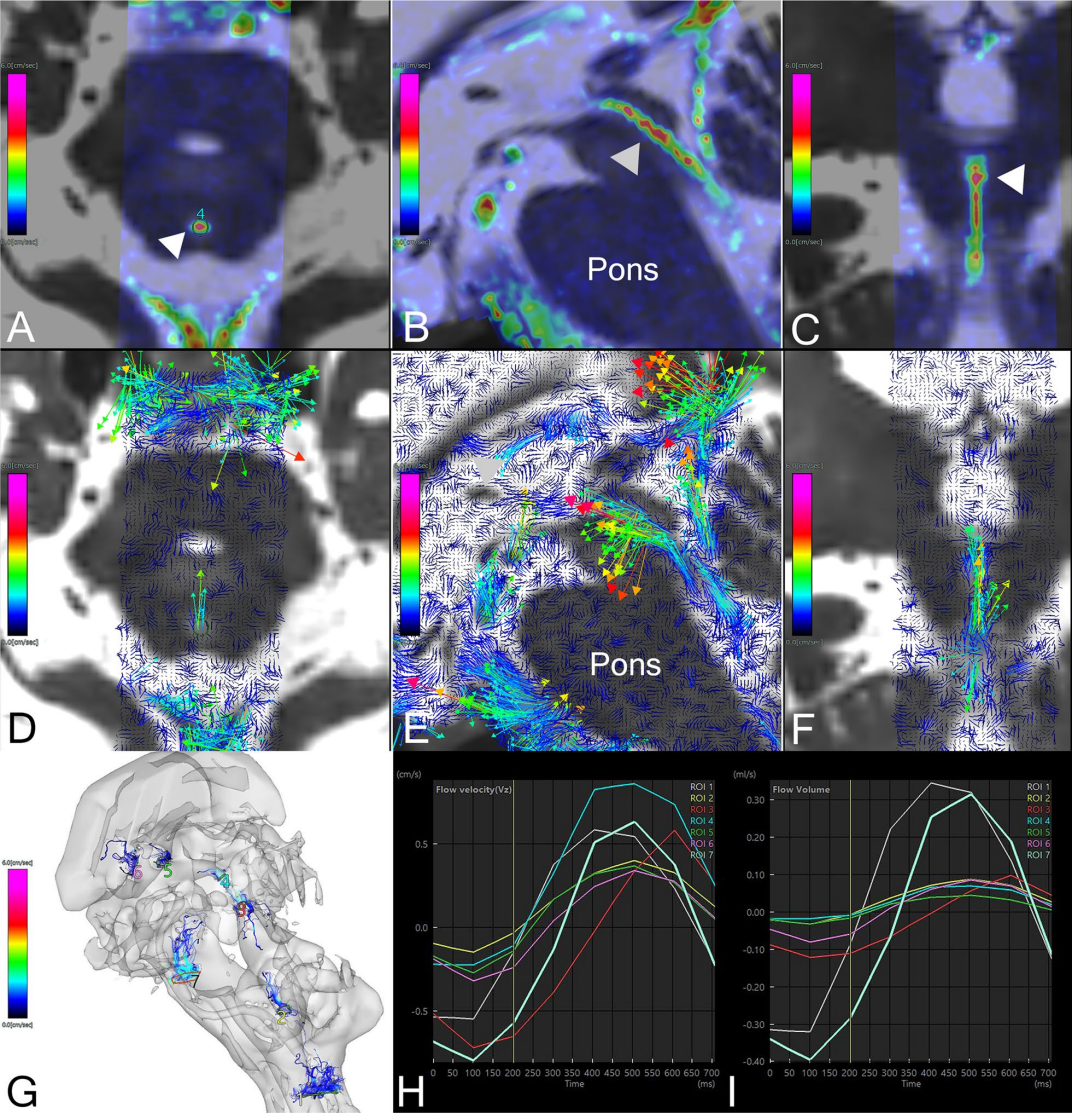
Four-dimensional flow magnetic resonance imaging. The axial (**A**) sagittal (**B**) and coronal (**C**) colormap views of 4D flow MRI analysis show the velocity magnitude of CSF movements around the cerebral aqueduct (white arrowheads) in a representative patient with Hakim’s disease (73 years old, female). The colored area indicates the area where the 3D flow velocity (cm/s) was measured on 4D flow MRI, and the color bar indicates the magnitude of the 3D velocity of reciprocating CSF movements: red for fast and blue for slow. The 2D sagittal views of flow vectors (**D**–**F**) show the CSF movements with direction and velocity. The color and size of arrows indicate the velocity magnitude. The 3D view of streamlines (**G**) shows the CSF movements thorough seven ROIs during a heartbeat (a movie of the streamlines was attached to the supplemental materials). The line graph **H** shows the flow velocity and the line graph **I** shows the flow volume at seven ROIs. The ROIs were manually drawn at seven points, including the dorsal aspects of the foramen magnum (ROI1), foramen of Magendie (ROI2), lower (ROI3) and upper (ROI4) parts of the cerebral aqueduct, left (ROI5) and right (ROI6) foramina of Monro, and prepontine cistern (ROI7). The line graphs show the temporal changes of flow velocity (**H**) and flow volume (**I**) at these ROIs during the cardiac cycle

**Fig. 3:**
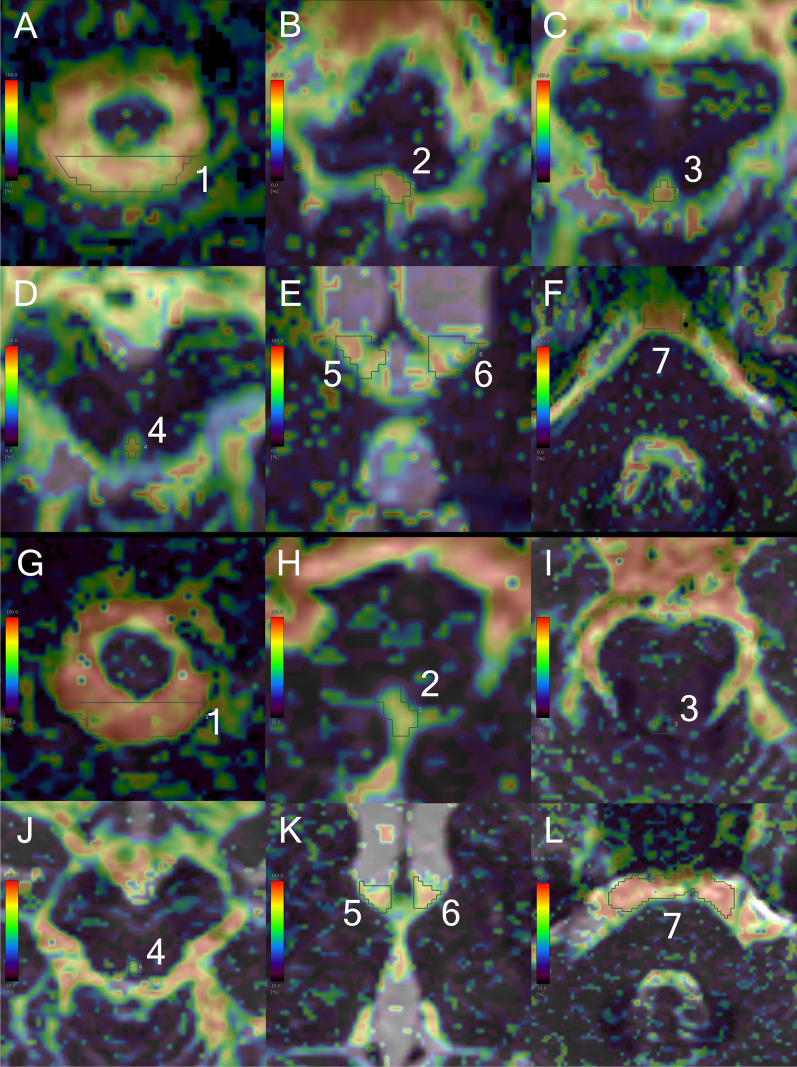
Intravoxel incoherent motion *f*-map in a patient with Hakim’s disease and healthy control. The upper **A**–**F** figures show the *f*-maps focused on seven ROIs in the same patient with Hakim’s disease as Fig. 2, and the lower **G**–**L** figures show the *f*-maps in a representative healthy volunteer with the same age and sex as the patient with Hakim’s disease (73 years old, female). The ROIs were manually drawn at seven points, including the dorsal aspects of the foramen magnum (ROI1) in **A** and **G**, foramen of Magendie (ROI2) in **B** and **H**, lower part of the cerebral aqueduct (ROI3) in **C** and **I**, upper part of the cerebral aqueduct (ROI4) in **D** and **J**, left (ROI5) and right (ROI6) foramina of Monro in **E** and **K**, and prepontine cistern (ROI7) in **F** and **L**. The color bar indicates the magnitude of the *f*-values on IVIM MRI: red for fast and blue for slow

The flow parameters on 4D flow MRI at seven ROIs (Fig. 2 and supplemental movie) were comprehensively assessed to determine their relationship with the mean *f*-values on IVIM MRI at the same location (Fig. 3). Table 2 shows the averages and ranges of stroke volume and VA measured by 4D Flow MRI and *f*-values measured by IVIM MRI at 7 ROIs. Based on these averages and ranges of these parameters, we were unable to determine which specific 4D flow MRI parameters correspond to the *f*-values optimally.

**Table 2:** Stroke volume (μL/min) and velocity amplitude (VA) (cm/s) on 4D flow MRI and *f* (%) on IVIM MRI

Region of interest	Stroke volume (μL/min)	VA (cm/s)	*f* (%)
Foramen magnum, mean ± SD	237 ± 145	1.33 ± 0.61	70.2 ± 16.6
(Range)	(40–1100)	(0.20–4.85)	(9.2–94.6)
Prepontine cistern, mean ± SD	162 ± 104	1.27 ± 0.72	87.3 ± 4.5
(Range)	(20–600)	(0.27–6.37)	(65.9–95.4)
Foramen of Magendie, mean ± SD	27.2 ± 23.3	0.49 ± 0.32	76.4 ± 8.4
(Range)	(0–150)	(0.10–2.14)	(36.6–93.4)
Lower part of cerebral aqueduct, mean ± SD	33.0 ± 29.6	0.78 ± 0.50	75.6 ± 7.5
(Range)	(0–220)	(0.14–3.40)	(45.5–89.9)
Upper part of cerebral aqueduct, mean ± SD	32.0 ± 35.2	0.94 ± 0.70	71.2 ± 11.1
(Range)	(0–170)	(0.07–3.53)	(30.6–89.7)
Left foramen of Monro, mean ± SD	34.3 ± 34.0	0.48 ± 0.26	74.7 ± 12.9
(Range)	(0–220)	(0.07–1.56)	(29.9–92.8)
Right foramen of Monro, mean ± SD	29.1 ± 24.7	0.41 ± 0.26	72.8 ± 14.2
(Range)	(0–120)	(0.05–1.67)	(8.6–92.5)

### Estimation of CSF movement from the f-values on IVIM MRI and fluid oscillation index (FOI)

**Fig. 4:**
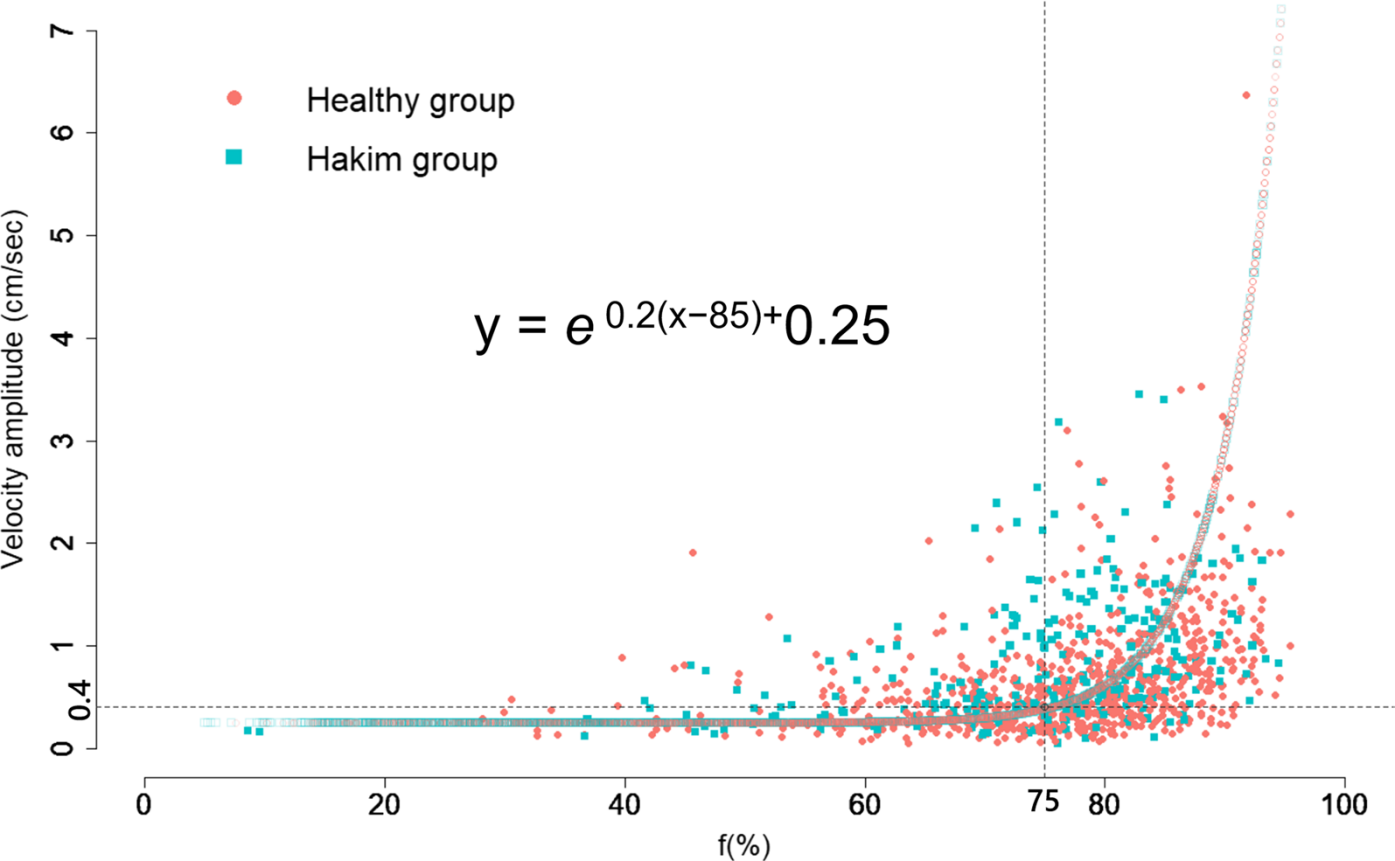
Distribution of *f* on IVIM MRI and velocity amplitude (VA) on 4D flow MRI. The scatter plot shows the relationship between the *f* (%) on IVIM MRI (x axis) and VA (cm/s) on 4D flow MRI (y axis). The color plots and lines indicate the healthy group (salmon pink) and patients with Hakim’s disease (sky blue). Filled-in points represent actual measurements, while hollowed-out points represent estimates calculated using the following formula: *e*^0.2(x−85)^ + 0.25. The dot vertical line indicates *f* = 75%, horizontal line VA = 0.4 cm/s

In order to elucidate the optimal 4D flow MRI parameters corresponding to *f* on IVIM MRI, a scatter plot was constructed to visualize the distribution of all *f*-values and VAs measured at 7 ROIs (Fig. 4).

**Fig. 5:**
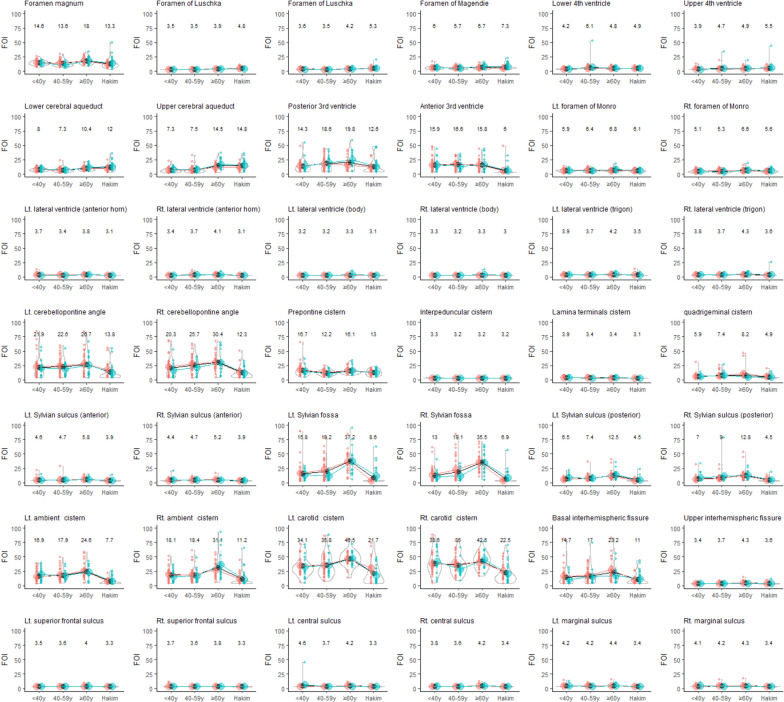
Distribution of fluid oscillation index (FOI) among healthy controls and Hakim patients. Each graph is a combination of violin plots for the distribution of the VA and line graphs for the mean volume in each age group of healthy volunteers and Hakim group stratified according to sex. Salmon pink indicates female, sky blue indicates male, and black indicates all. The vertical lines contain the volumes between the 25th and 75th percentiles. The mean FOI in each group is shown above the violin plot

Based on this distribution, it was deemed inappropriate to employ a linear regression model, and instead, fitting the data to a nonlinear exponential curve was considered to be the optimal approach. The exponential curve of *e*^0.2(x−85)^ + 0.25 was derived to best fit the distribution, providing a more accurate representation of the relationship between the *f*-values and VAs. From this regression equation, it was determined that VA corresponds to 0.4 cm/s when *f* is 75%. The utility of *f* surpassed that of VA in assessing flow oscillations when *f*-values were below 75%; conversely, VA proved more informative when *f* exceeded 75%. Consequently, in light of these findings, we introduced a novel index, FOI, calculated as VA (or estimated VA) × 10 + *f* × 0.02, which integrates these distinct parameters to quantitatively evaluate fluid oscillation. The distributions of FOIs across the entire intracranial space in the three age groups of healthy controls and the Hakim group are depicted in violin plots by sex (Fig. 5). FOIs were relatively large in the posterior subarachnoid space, foramen magnum, cerebral aqueduct, third ventricle, and Sylvian fossa, and very low in the lateral ventricles and the high parietal convexity part of the subarachnoid spaces.

### Relationship between measurements and indices specific to Hakim’s disease and fluid oscillation index (FOI)

**Fig. 6:**
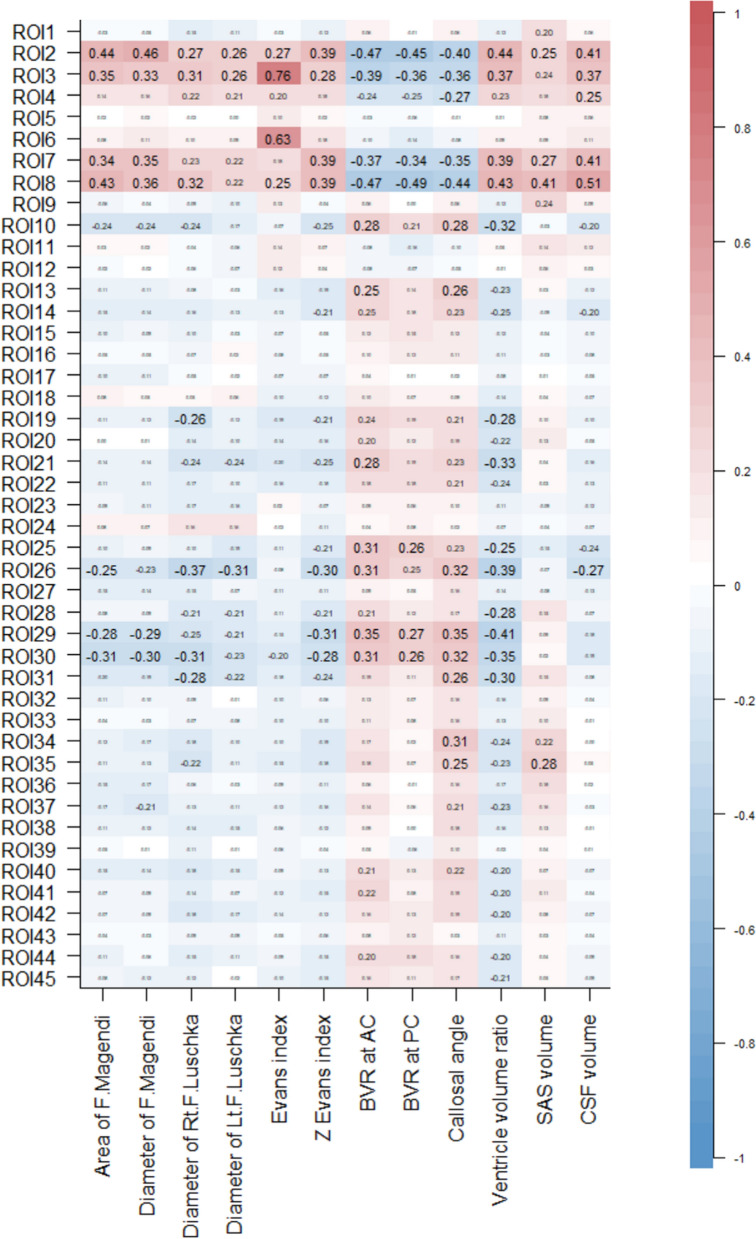
Correlation matrix between morphological parameters for Hakim’s disease and fluid oscillation index (FOI) in the entire intracranial CSF space. The correlation matrix is a comprehensive correlation analysis between parameters that indicate the morphological characteristics of Hakim’s disease and FOI. As shown in the color bar on the right, the red gradient indicates the strength of the positive correlation, whereas the blue gradient indicates the strength of the negative correlation. Furthermore, the numbers written inside were Pearson’s correlation coefficients. *F* foramen, *SAS* subarachnoid space, *CSF* cerebrospinal fluid. The numbers of regions of interests (ROIs) indicate the following regions: the foramen magnum (ROI1), right and left foramina of Luschka (ROI2 and 3), foramen of Magendie (ROI4), lower part of 4th ventricle (ROI5), 4th ventricle (ROI6), lower part of cerebral aqueduct (ROI7), upper part of cerebral aqueduct (ROI8), anterior part of 3rd ventricle (ROI9), posterior part of cerebral aqueduct (ROI10), right and left foramina of Monro (ROI11 and 12), right and left lateral ventricles (anterior horn ROI13 and 14, body ROI15 and 16, trigone ROI17 and 18, and inferior horn ROI19 and 20), right and left cerebellopontine angle (ROI21 and 22), prepontine cistern (ROI23), interpeduncular cistern (ROI24), lamina terminalis cistern (ROI25), suprasellar or chiasmatic cistern (ROI26), quadrigeminal cistern (ROI27), right and left ambient cisterns (ROI28 and 29), right and left carotid cisterns (ROI30 and 31), right and left Sylvian sulci (anterior ROI32 and 33, and posterior ROI36 and 37), right and left Sylvian fossae (ROI34 and 35), basal interhemispheric fissure (ROI38), upper part of interhemispheric fissure (ROI39), right and left superior frontal sulci (ROI40 and 41), right and left central sulci (ROI42 and 43), and right and left marginal sulci (ROI44 and 45)

We comprehensively examined the relationship between the morphological characteristics of Hakim’s disease and FOIs at 45 ROIs using correlation matrices. As shown in Fig. 6, the strongest correlations were observed between FOI at the left foramen of Luschka (ROI3) and the Evans index (*r*, 0.76; 95% CIs, 0.69 to 0.82; *p* < 0.001). The other indices related to Hakim’s disease including ventricular volume ratio (%) were significantly associated with the FOIs at the cerebral aqueduct (ROI7 and 8) and the bilateral foramina of Luschka (ROI2 and 3), rather than the foramen of Magendie (ROI4). The ROIs showing significant correlations with ventricular volume ratio (%) in Fig. 6 are summarized in Table 3.

**Table 3:** Pearson’s correlation coefficient (*r*) and 95% confidential intervals (95% CIs) for the relationship between ventricular volume ratio (%) and fluid oscillation index (FOI)

ROI number	ROI name	*r* (95% CIs)
ROI2	Rt. foramen of Luschka	0.44 (0.32–0.56)
ROI3	Lt. foramen of Luschka	0.37 (0.24–0.50)
ROI7	Lower end of cerebral aqueduct	0.39 (0.26–0.51)
ROI8	Upper end of cerebral aqueduct	0.43 (0.30–0.54)
ROI10	3rd ventricle (anterior part)	− 0.32 (− 0.45 to − 0.18)
ROI21	Rt. cerebellopontine angle	− 0.33 (− 0.46 to − 0.19)
ROI26	Suprasellar cistern	− 0.39 (− 0.51 to − 0.26)
ROI29	Lt. ambient cistern	− 0.41 (− 0.53 to − 0.28)
ROI30	Rt. carotid cistern	− 0.35 (− 0.47 to − 0.21)
ROI31	Lt. carotid cistern	− 0.30 (− 0.43 to − 0.15)

## Discussion

**Fig. 7:**
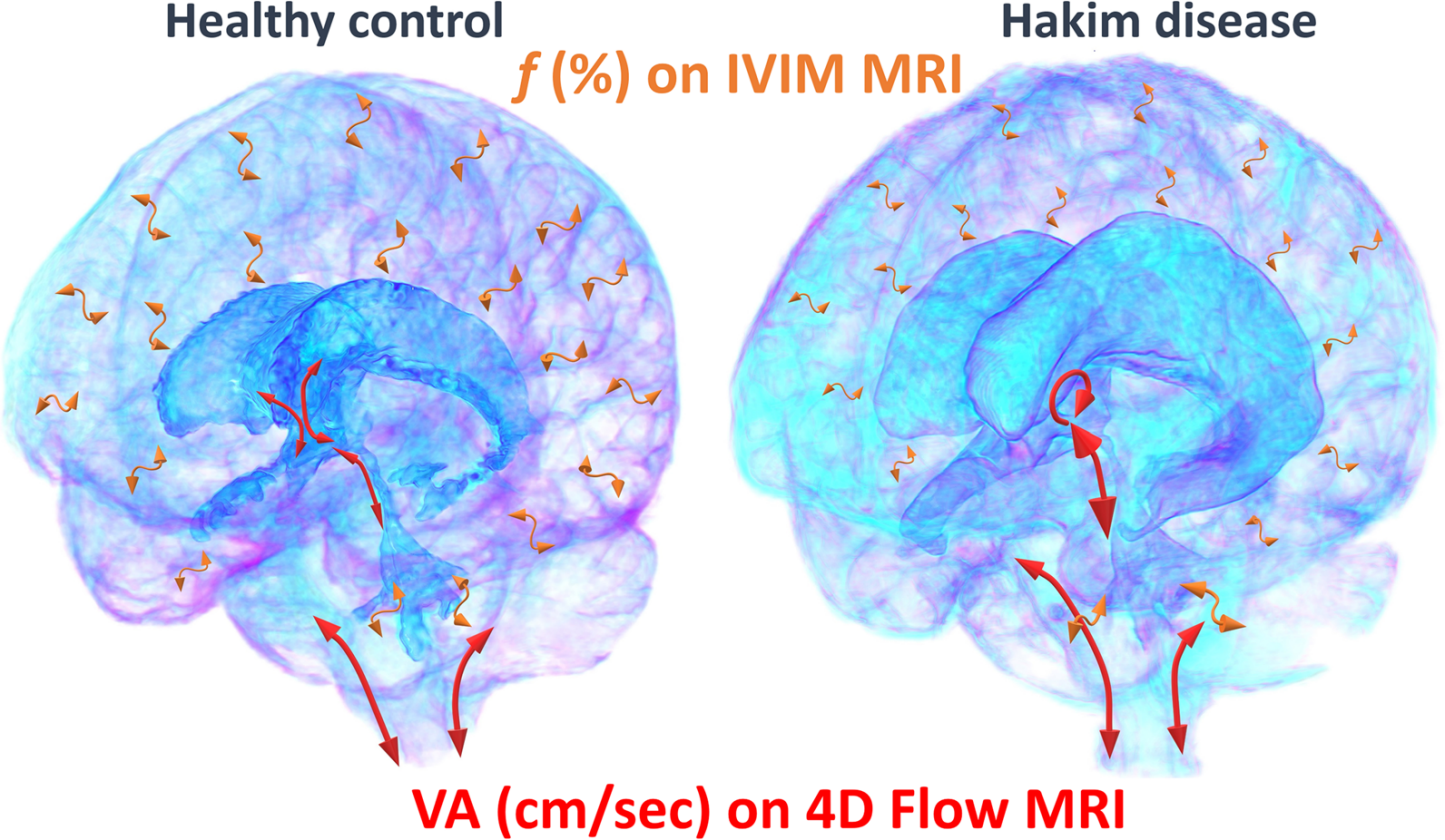
Illustration of CSF movement in a healthy control and a patient with Hakim’s disease. Compared to healthy controls, patients with Hakim’s disease have larger CSF repetitive movements measured by velocity amplitude (VA) on 4D Flow MRI at the cerebral aqueduct and vortex flow in the third ventricle (red arrows) and smaller CSF repetitive movements measured by *f* on IVIM MRI in the superficial subarachnoid space (orange arrows)

In this study, we proposed a novel method for estimating CSF motion throughout the entire intracranial CSF space by integrating flow velocity data from 4D flow MRI and *f* from IVIM MRI (Fig. 7). Although 4D flow MRI can three-dimensionally measure the velocity and direction of CSF motions, the range of velocity measurement depends on velocity encoding. Conversely, IVIM MRI could not directly measure the velocity and direction of CSF motions; however, it could visualize and quantify very small CSF motions that could not be measured using 4D flow MRI. Furthermore, IVIM MRI can provide an overview of all CSF movements driven by arterial pulsations, brain pulsations produced by the cerebral blood circulation, respiration, and coordinated directional beating of the motile cilia, except for head movements, because it does not require heartbeat or respiration synchronization. Therefore, we investigated whether the flow information from the two MRI methods could be used to simulate the overall intracranial flow.

From the distribution of VA on 4D Flow MRI and *f* on IVIM MRI in Fig. 4, VA, reciprocating bidirectional CSF motion or oscillation, should to be evaluated with a focus on VA rather than *f*, when the VA exceeds 0.4 cm/s. Conversely, when the VA is below 0.4 cm/s, *f* rather than VA would be better suited for evaluating reciprocating CSF motion. Based on the relationship between VA and *f*, a new FOI was created to comprehensively evaluate CSF oscillations by integrating both variables.

The major driving force of CSF movements is the pulsation of the brain due to cerebral circulation [2, 8, 20, 28, 39]. Brain pulsation generates and propagates waves in the CSF, and pressure gradients cause the CSF to move. Age-related changes in the intracranial environment require consideration of not only a decrease in brain volume [40, 41] but also an increase in CSF in the ventricles and subarachnoid space, a decrease in the total cerebral blood flow volume due to a decrease in brain volume [42], and a further decrease in pulsatile CSF movements due to a decrease in the cerebral blood flow volume. Compared with healthy aging volunteers, patients with Hakim’s disease had a significantly larger intracranial CSF volume, which is distributed disproportionately, such as ventricular dilatation, enlarged Sylvian fissure, and narrow sulci at high parietal convexity; these are collectively called DESH [32–34]. The reasons for the development of DESH, particularly the narrow sulci at high parietal convexity, remain to be fully elucidated. We observed that the FOI in the upper part of the cerebral aqueduct was similarly elevated in patients with Hakim’s disease and healthy elderly individuals aged ≥ 60 years. However, we found that the FOI in the third ventricle, particularly in the anterior part, was significantly lower in patients with Hakim’s disease compared to healthy elderly individuals. This difference may be attributed to alterations in CSF dynamics in the third ventricle, where a laminar to vortex flow pattern is established. Furthermore, we found the morphological features of DESH were significantly associated with the FOIs at the cerebral aqueduct and bilateral foramina of Luschka, rather than the foramen of Magendie. In our previous studies [6], we concluded that the enlargement of the foramen of Magendie with aging results in the transmission of CSF pulses into the ventricles, leading to ventricular enlargement. However, we have now discovered that fluid oscillations at the bilateral foramina of Luschka may be more closely associated with DESH formation, including ventricular enlargement, compared to those at the foramen of Magendie. CSF movements through the foramen magnum, which is the epicenter of CSF movements, play a dual role: to decrease pressure fluctuations associated with changes in the volume of blood inflow and outflow in the intracranial space during cerebral circulation and to regulate intracranial pressure during positional changes between supine and upright postures [19, 43]. This role requires an extensive spinal subarachnoid space contiguous to the lumbar region, where the CSF moves in and out of the skull base through the foramen magnum. Pulsatile CSF movements require energy according to the law of conservation of energy, and energy consumption can be minimized as much as possible by collecting the CSF near the epicenter, which is the foramen magnum. As CSF increases, it may accumulate close to the foramen magnum, where it is most mobile. Conversely, the supratentorial convexity region located farthest from the foramen magnum, where the CSF is least mobile, may become compressed, resulting in the characteristic morphology of DESH.

We observed an increase in pulsatile CSF motions in the Sylvian fossa, carotid cistern, and ambient cisterns, which contain the major intracranial arteries around the circle of Willis, with aging. However, there was a notable decrease in CSF oscillations in Hakim’s disease. Arterial pulsatility tends to increase with age due to aortic stiffness [44–48], but CSF oscillations due to arterial pulsation may be attenuated in Hakim’s disease with enlargement of the Sylvian fissure and basal cistern. Aging and various diseases such as hypertension and diabetes have been reported as risk factors for Hakim’s disease [8, 49–51], which is known to be caused by arteriosclerosis affecting the dilation and contraction of cerebral arteries [44]. Furthermore, patients with Hakim’s disease exhibit shorter and fewer perivascular spaces as well as severe periventricular hyperintensity on MRI compared to healthy controls [52]. Additionally, these abnormal features have been observed to improve after CSF shunt surgery [52]. These findings may be related to dysfunction of the glymphatic system [17, 18] in Hakim’s disease. However, the relationship between CSF flow and arterial wall pulsation has not been fully elucidated.

This study has some limitations. First, the distributions of sex and age in the healthy controls aged ≥ 60 years did not match those in the Hakim group. The mean age of the Hakim group was significantly higher than that of healthy controls aged ≥ 60 years. A sufficient number of older controls could have more clearly shown the difference in *f*-values between brains with Hakim’s disease and healthy aging brains. Second, the ROIs on 4D flow MRI and IVIM MRI were manually placed by a single researcher based on anatomical features; therefore, reproducibility was not ensured. We would like to enable automatic ROI placement in the application. Third, the correlation between the flow velocity parameters on 4D flow MRI and the mean *f*-values on IVIM MRI was not very strong. Additionally, VA = 0.4 cm/s was assumed to correspond to *f* = 75% in this study; however, these values may be influenced by sequence parameters such as the venc setting for 4D Flow MRI and the b-values setting for IVIM MRI. Finally, we applied a nonlinear exponential curve regression equation to estimate VA from the mean *f*-values on IVIM MRI. In the future, age-related changes in CSF dynamics in the entire intracranial CSF space should be simulated using advanced computational fluid dynamics data assimilation of flow velocity data obtained using 4D flow MRI and IVIM MRI to elucidate the mechanism of disproportionate CSF distribution, including DESH, in Hakim’s disease. Data assimilation using computational fluid dynamics and lattice models is most effective in situations where complex fluid motions must be predicted.

## Conclusions

We comprehensively investigated the CSF dynamics in healthy individuals with ages ranging from their 20 s to 80 s and patients with Hakim’s disease (iNPH) by integrating 4D flow MRI and IVIM MRI. We estimated the fluid oscillations in the entire intracranial CSF space using a novel index, the FOI, which combined VA derived from 4D flow MRI with *f*-values derived from IVIM MRI. In addition, we found the ventricle volume ratio and morphological features of Hakim’s disease were significantly associated with the FOIs at the cerebral aqueduct and bilateral foramina of Luschka, rather than the foramen of Magendie. Furthermore, FOIs at the cerebral aqueduct and bilateral foramina of Luschka were elevated in both Hakim’s disease and healthy controls aged ≥ 60 years. The computational simulation by estimating CSF motion across the entire intracranial CSF space on both 4D flow MRI and IVIM MRI could help explore the underlying mechanisms of disproportionate expansion of the ventricles and subarachnoid spaces in Hakim’s disease.

### Supplementary Information

Supplementary Material 1.

## Data Availability

The data, including clinical information, collected and analyzed in this study will only be available if the Ethics Committees approve new participation in the collaborative research.

## Abbreviations

BVR: Brain per ventricle ratio
CSF: Cerebrospinal fluid
DESH: Disproportionately enlarged subarachnoid space hydrocephalus
DWI: Diffusion weighted image
f: Fraction of incoherent perfusion
FOI: Fluid oscillation index
IVIM: Intravoxel incoherent motion
PC: Phase-contrast
ROI: Region of interest
SD: Standard deviation
VA: Velocity amplitude
